# *MIP/Aquaporin 0* Represents a Direct Transcriptional Target of PITX3 in the Developing Lens

**DOI:** 10.1371/journal.pone.0021122

**Published:** 2011-06-17

**Authors:** Elena A. Sorokina, Sanaa Muheisen, Nevin Mlodik, Elena V. Semina

**Affiliations:** 1 Department of Pediatrics and Children's Research Institute, Medical College of Wisconsin and Children's Hospital of Wisconsin, Milwaukee, Wisconsin, United States of America; 2 Department of Cell Biology, Neurobiology and Anatomy, Medical College of Wisconsin, Milwaukee, Wisconsin, United States of America; University of Illinois at Chicago, United States of America

## Abstract

The PITX3 *bicoid*-type homeodomain transcription factor plays an important role in lens development in vertebrates. *PITX3* deficiency results in a spectrum of phenotypes from isolated cataracts to microphthalmia in humans, and lens degeneration in mice and zebrafish. While identification of downstream targets of PITX3 is vital for understanding the mechanisms of normal ocular development and human disease, these targets remain largely unknown. To isolate genes that are directly regulated by PITX3, we performed a search for genomic sequences that contain evolutionarily conserved *bicoid*/PITX3 binding sites and are located in the proximity of known genes. Two *bicoid* sites that are conserved from zebrafish to human were identified within the human promoter of the major intrinsic protein of lens fiber, *MIP/AQP0*. *MIP/AQP0* deficiency was previously shown to be associated with lens defects in humans and mice. We demonstrate by both chromatin immunoprecipitation and electrophoretic mobility shift assay that PITX3 binds to *MIP/AQP0* promoter region in vivo and is able to interact with both *bicoid* sites in vitro. In addition, we show that wild-type PITX3 is able to activate the *MIP/AQP0* promoter via interaction with the proximal *bicoid* site in cotransfection experiments and that the introduction of mutations disrupting binding to this site abolishes this activation. Furthermore, mutant forms of PITX3 fail to produce the same levels of transactivation as wild-type when cotransfected with the *MIP/AQP0* reporter. Finally, knockdown of *pitx3* in zebrafish affects formation of a DNA-protein complex associated with *mip1* promoter sequences; and examination of expression in *pitx3* morphant and control zebrafish revealed a delay in and reduction of *mip1* expression in *pitx3*-deficient embryos. Therefore, our data suggest that PITX3 is involved in direct regulation of *MIP/AQP0* expression and that the alteration of *MIP/AQP0* expression is likely to contribute to the lens phenotype in cataract patients with *PITX3* mutations.

## Introduction

The PITX3 *bicoid*-related homeodomain transcription factor represents an important regulator of lens development in vertebrates. Mutations in *PITX3* result in congenital cataracts, anterior segment mesenchymal dysgenesis (ASMD), Peter's anomaly, and microphthalmia in humans [1]–[6]. Deletions within the *Pitx3* promoter region in mice produce the *aphakia* phenotype, which is characterized by small eyes lacking a lens [7], [8]. In lower vertebrates (zebrafish and frog), *pitx3* was shown to be essential to normal lens and retina formation [9]–[13]. Knockdown of pitx3 protein in zebrafish embryos via translational morpholino results in small eyes, lens degeneration, misshapen head and reduced jaw and fins [9], [10], [12]. In vertebrates, expression of *Pitx3/pitx3* is first detected in the lens placode and then the lens vesicle; early expression is observed in the lens epithelial cells and primary fibers while later expression is restricted to the equator regions of the developing lens [1], [14].

Despite its vital importance for eye development, little is currently known about the ocular function of *PITX3/Pitx3* and its downstream targets. Expression of several genes/proteins was found to be altered in the lenses of *Pitx3*-deficient mice. Some early reports demonstrated that expression of β- and γ -crystallins is completely absent at developmental stages 10–18 days as well as in newborn *aphakia* mice [15]–[17]. Two recent publications provided additional data on this matter; Ho and colleagues detected precocious activation of both β- and γ-crystallins in the eyes of 10.5–11.5-dpc *Pitx3*–knockout mice [18] while Medina-Martinez and coauthors reported deregulation of crystallin expression in *aphakia* mice with α- and β- crystallin expression being reduced at both transcript and protein levels and γ –crystallin expression being downregulated at the protein level [19]. In addition to crystallins, expression of the transcription factors *Foxe3* [18]–[20] and *Prox1* [19] as well as the cell cycle regulator *p57KIP2* [19] were found to be affected in *Pitx3*-deficient animals, which seems more likely to be related to the overall abnormal lens development in *aphakia* mice rather than direct involvement of Pitx3 in transcriptional regulation of these genes [19].

PITX3 belongs to the PITX family of *bicoid*-type homeodomain-containing proteins that regulate expression of other genes during development and, possibly, in adult organisms. Other members of this family were shown to be involved in developmental disorders such as idiopathic clubfoot [*PITX1*; 21] and Axenfeld-Rieger syndrome [*PITX2*; 22]. PITX factors are known to interact with *bicoid*-type DNA sequences and to regulate downstream gene expression through these interactions [23]–[28]. PITX factors are primarily known as activators of transcription, though they may also act as repressors [29], [30]. Several transcriptional targets of PITX homeoproteins have been identified and *bicoid* sequences located in the regulatory regions of these downstream genes were shown to mediate these interactions; two or more *bicoid* sites were found in some promoters [25], [26], [28], [31], [32], although a single *bicoid* element was demonstrated to be sufficient in several other cases [24], [33]–[35]. Interspecies conservation of *bicoid* sequences has been reported for some promoters [26], [32]. Preservation of regulatory sequences is frequently observed for developmental genes which demonstrate a conserved expression pattern; therefore, identification of regulatory sequences represents a useful tool in uncovering genetic pathways [36], [37].

In order to isolate downstream targets of the PITX3 homeodomain transcription factor we performed a search for evolutionarily conserved non-coding sequences containing *bicoid* sites and located in proximity to known genes, therefore potentially interacting with PITX3 to regulate expression of that gene. As a result, we identified two *bicoid* sites located in the promoter of *Major Intrinsic Protein of lens fiber* (*MIP*) or *Aquaporin 0* (*AQP0*) that are conserved between human, mouse, zebrafish and several other species. We further demonstrated that PITX3 is able to specifically interact with the identified sequences both *in vitro* and *in vivo* and to transactivate gene expression as a result of this interaction. In addition to this, expression of *mip1* was found to be altered in *pitx3* deficient zebrafish morphants. Our data suggest that PITX3 is involved in direct regulation of *MIP/AQP0* expression and provide new insight into the PITX3 pathway as well as mechanisms of lens development.

## Materials and Methods

### Ethics statement

The study was carried out in accordance with the recommendations in the Guide for the Care and Use of Laboratory Animals of the National Institutes of Health. The protocol was approved by the Institutional Animal Care and Use Committee at the Medical College of Wisconsin (protocol number AUA00000352).

### In silico analysis

ECR Browser web-based tool (http://ecrbrowser.dcode.org) was used to identify conserved paired *bicoid* sites in the promoters/intronic regions of genes with known expression/function. Paired comparison of human and mouse genomes was performed using the following parameters: presence of two conserved *bicoid* sites with distance between the sites not to exceed 650-bp. Secondary analysis of identified regions for sequence conservation was performed using the UCSC Genome Brower multiple alignment module (http://genome.ucsc.edu) as well as the BLAST tool (http://blast.ncbi.nlm.nih.gov), including examination of the corresponding genes in lower vertebrates when available.

### Cell culture

Human lens epithelial cells (B3) and human embryonic kidney cells (293HEK) were obtained from ATCC (Manassas, VA). B3 cells were cultured in MEM medium (Invitrogen; Carlsbad, CA) supplemented with heat-inactivated 20% fetal calf serum (FBS), glutamine, sodium pyruvate to a final concentration of 1 mM, non-essential amino acids and antibiotic-antimycotic (Invitrogen; Carlsbad, CA). 293HEK cells were maintained in DMEM medium containing 10% FBS, glutamine and antibiotic-antimycotic solution.

### Electrophoretic Mobility Shift Assay (EMSA)

Nuclear extracts were prepared from B3 cells transiently transfected with PITX3-pcDNA3.1 vector with CelLytic NuCLEAR extraction kit (Sigma, St. Louis, MO). Cells were harvested after 48 hours with a cell scraper and the compact cellular pellet was re-suspended in 5 volumes of Hypotonic lysis buffer (10 mM HEPES, pH 7.9, 1.5 mM MgCl_2_, 10 mM KCl) with Protease inhibitor cocktail (Sigma; St. Louis, MO). After 15 minutes of incubation on ice, Igepal CA-630 was added to a final concentration 0.6%, then the cells were vortexed and spun down for 30 seconds at 10000 g. Crude nuclear pellet was extracted with about 2/3 of the original packed cell volume of Extraction buffer (20 mM HEPES, pH 7.9, 1.5 mM MgCl_2_, 0.42 M NaCl, 0.2 mM EDTA, 1 mM DTT and 25% Glycerol) in the presence of protease inhibitors for 30 minutes on ice. 32-mer 5′-GGAGAAAGGCTTCTAATCCCTGGGAACTAAAG oligonucleotide spanning region −533/−502 from transcriptional start site (tss) of *MIP/AQP0* promoter, 32-mer 5′-CTGCCCCTCCCAGGGATTAAGAGTCCTCTATA corresponding to the promoter sequence −71/−40 and their complement oligonucletides as well as both sets of oligonucleotides with TAATCC (GGATTA) *bicoid* sites replaced by TAATTT (AAATTA) (see above) were labeled with Biotin 3′ End DNA Labeling kit (PIERCE) and annealed. Electrophoretic mobility shift assays (EMSA) were performed with LightShift Chemiluminescent EMSA kit (Pierce; Rockford, IL) in accordance with the manufacturer's protocol and using 50 ng/µl of Poly(dI-dC), 20 fmol of labeled DNA and 2 µl of nuclear extracts. After 20 minutes of incubation at room temperature, reactions were either diluted with 5× Loading buffer or further incubated for 30 minutes in presence of 1 µg of polyclonal Pitx3 antibody for supershift assay. Binding reactions and free probe were run on 5% native polyacrylamide gel in 0.5× TBE buffer.

For EMSA experiments performed using whole zebrafish embryo nuclear extracts, the 32-mer and its compliment corresponding to the region from −44 to −76 of zebrafish *mip1* promoter were utilized: 5′-CAA TTC AGC CAA AGG ATT ACA GTG TCA CAG AG. In addition to this, both sets of oligonucleotides were made with TAATCC (GGATTA) *bicoid* sites replaced by TAATTT (AAATTA) to be used as a control for *bicoid* site binding specificity. Nuclear extracts were generated from sixty 48-hpf zebrafish pitx3 morphant or wild-type embryos that demonstrated normal body length and morphology; the preparation was carried out as described above except for that the embryos were first grinded with glass tissue homogenizer equipped with type B pestle in hypotonic detergent-less lysis buffer to assist nuclei release. Binding reaction was performed in the presence of 2.5% glycerol, 5 mM MgCl2 and 0.05% NP-40 in addition to the buffer composition described above; 3 µl of extract was used in each binding reaction. Five embryos from each group were analyzed for *pitx3* transcript presence to verify the degree of morpholino-mediated knockdown.

### Chromatin immunoprecipitation (ChIP)

ChIP was performed with ChIP-IT enzymatic kit or ChIP-IT Express enzymatic kit (Active Motif; Carlsbad CA) according to manufacturer recommendations.

B3 human lens epithelial (HLE) cells were grown in 100 mm tissue culture dish to 90–95% confluence and utilized for ChIP assays; experiments were performed using native untransfected cells as well as cells transfected with PITX3 expression plasmids. Cells were transfected with 7.5–10 µg of pcDNA3.1_PITX3_FLAG or empty pcDNA3.1 plasmid and cross-linked after 48 hours with 1% formaldehyde for 10 minutes at room temperature with agitation. Following this, the monolayers were washed with 125 mM of glycine and lysed. The nuclear pellet was resuspended in digestion buffer and DNA was sheared with Enzymatic shearing cocktail for 10 minutes at 37°C in a water bath. The resulting fragments ranged between 200- and 1000-bp in size. The quality of chromatin was verified in a control experiment of immunoprecipitation with the Polymerase II antibody followed by PCR with primers specific for the GADPH promoter. Only those chromatin preparations that demonstrated significant enrichment in these control experiments were used in further analysis. Immunoprecipitation was performed with 2 µg of Pitx3, FLAG or control IgG antibody overnight in a cold room and, after washing and de-crosslinking, the precipitated DNA was analyzed by PCR. Goat polyclonal PITX3 (N-20) and normal goat IgG were purchased from Santa Cruz Biotechnology, Inc. (Santa Cruz, CA) and FLAG-M2 mouse monoclonal antibody from Sigma (St. Louis, MO). For PCR amplification of *MIP/AQP0* promoter, the following primers were utilized: set 1 (spanning region −110/+95) forward, 5′-GCTGTGAAGGGGTTAAGAGG-3′ and reverse 5′-GAGGGTGGCAAAGAACTCAG-3′ and set 2 (spanning region −473/−275) forward, 5′-CTGAACCCCACTCCTTACCA-3′ and reverse, 5′- TCTGCCCTTCTGTGTGTGTC-3′. For control experiments, the following primers were used: GAPDH forward 5′- TACTAGCGGTTTTACGGGCG-3′ and reverse 5′-TCGAACAGGAGGAGCAGAGAGCGA-3′, product = 166 bp (provided as positive control as part of ChIP-IT Express Enzymatic kit (Active Motif; Carlsbad, CA); forward 5′-ATGGTTGCCACTGGGGATCT-3′ and reverse 5′-TGCCAAAGCCTAGGGGAAGA-3′, product = 174 bp (provided as negative control as part of ChIP-IT Express Enzymatic kit, Active Motif, Carlsbad CA). The PCR reactions were repeated at least three times using precipitated DNA from independent chromatin immunoprecipitation experiments. Quantification of ChIP PCR products was performed by densitometry using the ImageJ program developed by Dr. Rasband, NIH (http://rsbweb.nih.gov/ij/). The measurements obtained for ChIP PCR results were normalized by input DNA and expressed as percent of its value. Data from at least three independent experiments were combined to calculate mean and standard deviation. Statistical significance was determined using the homoscedastic Student's t-test with two-tailed distribution.

### Expression and reporter plasmids

*PITX3* wild type, *PITX3-WT*, and mutant expression constructs, *PITX3-K111E, PITX3-S13N and PITX3- G219f*, were previously described [27]. To produce the *MIP656-bcd1,2* reporter plasmid, a 656-bp fragment containing 597-bp of upstream and 59-bp of downstream sequence from the transcriptional start site (tss) of *MIP/AQP0* was amplified by PCR and cloned into the pCRII-TOPO vector (Invitrogen; Carlsbad, CA) and then subcloned into basic pGL3 luciferase reporter vector (Promega, Madison, WI). The transcriptional start site (tss) of *MIP/AQP0* was designated based on the ENST00000257979 entry in Ensemble Database. Site-directed mutagenesis was performed with QuikChange II Site-Directed mutagenesis kit (Stratagene, La Jolla, CA), oligonucleotide 5′-CTCAGCCTGCCCCTCCCAGAAATTAAGAGTCCTCTATAAA-3′ and its complement for the proximal *bicoid* site and oligonucleotide 5′-CTAGCCAATGGGAGAAAGGCTTCTAATTTCTGGGAACTAAAGAATT-3′ and its compliment for the distal site on *MIP/AQP0* promoter. Therefore, in both *bicoid* sites the consensus recognition sequence TAATCC was replaced by TAATTT and three additional constructs were produced: *MIP656-bcd1* (carrying mutant *bcd2* site), *MIP656-bcd2* (carrying mutant *bcd1* site) and *MIP656-bcd0* (carrying mutations in both *bicoid* sites). All constructs were verified by sequencing.

### Reporter assays

Human embryonic kidney cells (293HEK) were plated in 24-well plates and transfected using Lipofectamine 2000 (Invitrogen; Carlsbad, CA) according to the manufacturer's protocol. Equimolar amounts of basic pGL3 reporter plasmid (100 ng) and *MIP656* wild-type and mutant reporters (114 ng) were used. Each cotransfection included 300 ng of effector (*PITX3* wild type and mutant expression constructs) and 60 ng of β-galactosidase in pcDNA3.1 vector (internal control for efficiency of transfection); the total DNA amount was kept the same in all transfections by adding empty pcDNA3.1 vector when needed. Cells were harvested after 48 hours; luciferase and β-galactosidase activities were determined using Luciferase assay and Enzyme Assay systems (Promega, Madison, WI), respectively. Every experiment was performed at least three times in triplicate. Student's paired t-Test with a one-tailed distribution was utilized to determine the statistical significance of any differences in activity level.

### Zebrafish care and morpholino injections

Zebrafish (*Danio rerio*) were maintained on a 14-hour light/ 10-hour dark cycle. The embryos were obtained by natural spawning and maintained at 28.5°C. The *pitx3* morpholino, 5′-AGGTTAAAATCCATCACCTCTACCG-3′, that was previously reported [9] or control morpholino (Gene Tools, Philomath, OR) were suspended at 250 µM in injection buffer [0.1% (w/v) phenol red (Sigma) in 0.3× Danieau buffer (17 mM NaCl, 2 mM KCl, 0.12 mM MgSO_4_, 1.8 mM Ca(NO3)_2_ and 1.5 mM HEPES, pH 7.6) and 19.2 ng was injected into zebrafish embryos immediately after fertilization at the 1–2 cell stage. Microinjections were performed using the Nanoject II injector (Drummond Scientific, Broomall, PA). Embryos were incubated at 28.5°C in 0.2 mM 1-phenyl-2-thiourea (PTU) to inhibit pigment formation and anesthetized with 0.05% Tricane before imaging. The developmental stage was determined by time (hours post fertilization (hpf)) and by morphological criteria [38]. Nikon SMZ 1500 and Zeiss M2 Discovery microscopes were utilized for embryo imaging.

### RNA isolation, RT-PCR and in situ hybridization

For RNA isolation, the embryos were homogenized in TRI reagent (Sigma) in the presence of glycogen and processed using a standard extraction protocol. The cDNA was generated using equal amounts of RNA for every sample and SuperScript III First-Strand Synthesis system (Invitrogen, Carlsbad, CA) according to manufacturer recommendations. Semi-quantitative PCR was performed using gene-specific oligonucleotides for *mip1*, exon_1F, 5′- CTCCCAGATGTCCCTGTTTC-3′, and exon_2R, 5′- CATACTGATGCCAGGCTGAA-3′, (PCR product = 148 bp); for *pitx3*, exon_1F, 5′-CTCCACTAGACCGGGATTCA-3′, and exon_3R, 5′- AAAGGTGGCTTCCAGTTCCT-3′ (PCR product = 276 bp); and for *β-actin*, exon2F, 5′-GAGAAGATCTGGCATCACAC-3′ and exon_3R, 5′-ATCAGGTAGTCTGTCAGGTC-3′ (PCR product = 323 bp). The PCR conditions were as follows: initial denaturation at 94°C for 3 min followed by 23–32 cycles of 94°C for 20 seconds, 59°C for 30 seconds and 72°C for 30 seconds and final extension at 72°C for 7 min. The number of cycles was optimized to maintain PCR reaction in linear range.

To construct an antisense riboprobe for *in situ* hybridization experiments, a 368-bp fragment specific to zebrafish *mip1* transcript was generated using the following primers, forward, 5′-CTGCAGGACATGCTCATCAC-3′ and reverse, 5′-GGCTGCAAAAAGTCAACAGA-3′, and inserted into pCRII-TOPO plasmid vector. An antisense RNA probe was generated using DIG RNA Labeling Kit (Roche Applied Science, Indianapolis, IN) following manufacturer recommendation; *in situ* hybridization was performed as previously described [39].

## Results

### Genome search for regulatory regions containing conserved PITX3 binding sites identifies MIP/AQP0 promoter

Examination of the ECR Browser web-based tool for clusters of PITX3 binding sites conserved between different species yielded a total of 976 genomic regions: 511 sequences were found inside of intergenic regions, 454 elements were located within genes (309 in intronic, 90 in coding and 55 in untranslated regions), and only 11 were positioned within 1500 bp from a transcriptional start site. The eleven identified promoter regions were subjected to a secondary analysis of sequence conservation that included examination of the corresponding genes in lower vertebrates when available. One sequence demonstrated the strongest level of conservation of *bicoid* sites across multiple species- the promoter region of the gene encoding for the major intrinsic protein of lens fiber or aquaporin 0 (*MIP/AQP0*).

**Figure 1:**
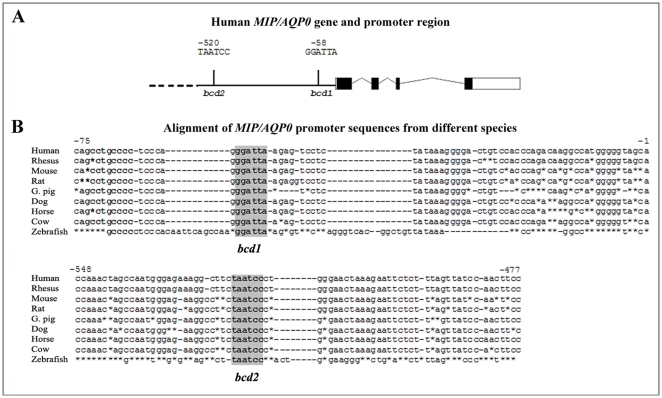
Human *MIP/AQP0* genomic region and *bicoid* elements. **A.** Schematic representation of the *MIP/AQP0* gene and promoter region; *bcd1* and *bcd2* sites are indicated. **B.** Multiple species alignment of genomic sequences surrounding the *bcd1* and *bcd2* sites (highlighted in grey). GenBank accession numbers are as follows: NT_029419.12 (*Homo sapiens*); NC_007868.1 (rhesus, *Macaca mulatta*), NT_039500.7 (mouse, *Mus musculus*), NC_005106.2 (rat, *Rattus Norvegicus*), NC_006592.2 (dog, *Canis lupus familiaris*), AAKN02014837.1 G.Pig, *Cavia porcellus*), NC_009149.2 (horse, *Equus caballus*), NC_007303.4 (cow, *Bos taurus*), NC_007134.4 (zebrafish, *Danio rerio*).

The *MIP/AQP0* promoter region contains two *bicoid* sites separated by 456 base pairs at positions −58 (*bcd1*) and −520 (*bcd2*) from the transcriptional start site. Alignment of *MIP/AQP0* promoters from different species demonstrates high conservation of both *bicoid* sites in nine mammalian/vertebrate species from human to zebrafish (Figure 1). Zebrafish (*Danio rerio*) has two orthologs of the human *MIP/AQP0* gene designated *mip1* and *mip2* [40]. The promoter sequence/structure of zebrafish *mip1* appears to be more similar to mammalian species showing conservation for both *bicoid* sites (positions −59 (*bcd1*) and −442 (*bcd2*)) and surrounding sequence (Figure 1).

Conservation of the *bicoid* sites in *MIP* promoters of different species points to the potential importance of these sequences in the regulation of *MIP/AQP0* expression.

### The conserved bicoid sequences within the MIP/AQP0 promoter are capable of binding PITX3

**Figure 2:**
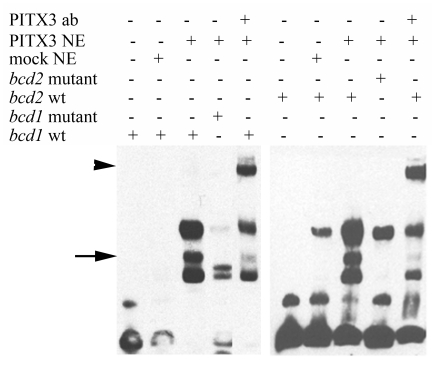
Electrophoretic mobility shift assays (EMSA) demonstrate interaction between PITX3 and *bcd1* and *bcd2* sites. EMSA performed with *bcd1* and *bcd2* oligonucleotides. DNA-PITX3 complexes are indicated with a full arrow; supershifts are shown with an arrowhead. ab = antibody, NE = Nuclear extracts, wt = wild type.

We first performed electrophoretic mobility shift assay (EMSA) to examine whether these putative *bicoid* sequences are able to bind PITX3 *in vitro*. Nuclear extracts were isolated from human lens epithelial cells transiently transfected with either a *PITX3* expression plasmid or an empty pcDNA vector and incubated with labeled oligonucleotides containing the TAATCC motif and 13-bp of flanking sequences on either side of each *bicoid* site. The samples derived from *PITX3*-enriched nuclear extracts produced clearly visible shifts with both probes which were not observed with samples prepared from mock-transfected cells (Figure 2). The PITX3-DNA complexes were further verified by addition of PITX3 polyclonal antibody, which resulted in reduction of the intensities of the shifted bands and formation of supershifts (Figure 2). In addition to this, the specificity of binding was confirmed by EMSA analysis using modified oligonucleotides carrying a 2-nt mutation within the *bicoid* sites: the TAATCC sequence was replaced with TAATTT in both probes to abolish PITX3 binding [27]. Mutations in the *bicoid* sites resulted in the disappearance of protein-DNA complexes, confirming that these bands are the product of specific PITX3-DNA interactions (Figure 2).

These results demonstrate that PITX3 is capable of binding to both *bicoid* sites located in the human *MIP/AQP0* promoter *in vitro*.

### PITX3 interacts with MIP/AQP0 promoter in human lens epithelial cells in vivo

To investigate whether PITX3 interacts with the *MIP/AQP0* promoter *in vivo*, we performed chromatin immunoprecipitation (ChIP) assays. Native untransfected human lens epithelial (HLE) cells or HLE cells following transfection with pcDNA3.1_PITX3_FLAG expression plasmid or pcDNA3.1 empty vector were used in these experiments.

**Figure 3:**
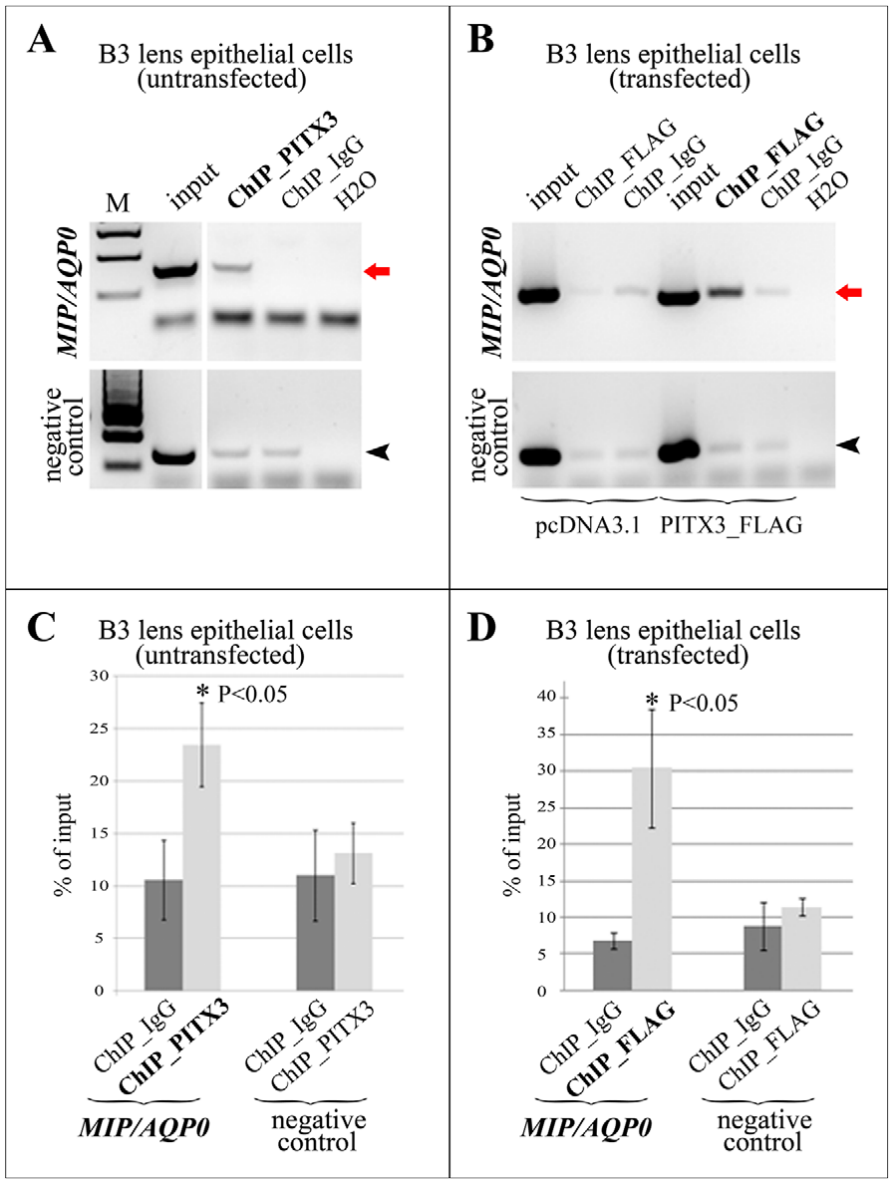
*MIP/AQP0* region demonstrates enrichment in chromatin immunoprecipitation experiments with PITX3 or FLAG antibody. **A.** Endogenous PITX3 is bound to the proximal *MIP/AQP0* promoter in human lens epithelial (HLE) cell cultures. Chromatin immunoprecipitation assays were performed using untransfected HLE cells and human PITX3 or IgG (control) antibodies. The samples were analyzed by semi-quantitative PCR using *MIP/AQP0* proximal promoter- specific primers and negative control primers. Please note robust amplification of *MIP/AQP0* promoter region from ChIP sample precipitated with PITX3 but not with control IgG antibody (red arrow) and equal levels of DNA amplification for negative control region in both samples (black arrowhead). **B.** PITX3_FLAG is bound to proximal *MIP/AQP0* promoter in HLE cell cultures following transfection with PITX3-FLAG expression plasmid. HLE cells were transfected with either PITX3-FLAG expression plasmid or control pcDNA3.1 expression vector. ChIP assays were performed with FLAG-M2 or control IgG antibody. The ChIP samples were analyzed by semi-quantitative PCR as described in A. Please note enrichment of *MIP/AQP0* promoter region in ChIP sample obtained from PITX3-FLAG transfected cells and precipitated with FLAG antibody in comparison to FLAG-precipitated ChIP sample obtained from pcDNA3.1 transfected cells as well as IgG-precipitated ChIP sample obtained using either PITX3-FLAG or pcDNA3.1 transfected cells (red arrow). In addition to this, amplification of negative control region demonstrated similar levels across all samples (black arrowhead). **C** and **D.** Statistical analysis of multiple semi-quantitative PCR/ChIP experiments performed in untransfected (C) and transfected HLE cells (D) as described in A and B, correspondingly. Presence of *MIP/AQP0* promoter or negative control region DNA in various ChIP samples was evaluated by semi-quantitative PCR followed by densitometric analysis and expressed as a percentage of input values; mean and standard deviation for at least 3 independent experiments were calculated and analyzed by Student's t test. Please note statistically significant enrichment for *MIP/AQP0* promoter region DNA precipitated with PITX3 (C) or FLAG (D) antibody in comparison to control IgG-precipitated chromatin in HLE untransfected (C) or transfected (D) cells. IgG = normal mouse IgG; PITX3 = PITX3 polyclonal antibody; FLAG = anti-FLAG monoclonal antibody.

Immunoprecipitations with PITX3 antibody that used nuclear extracts from native untransfected HLE cells resulted in enrichment of *MIP/AQP0* promoter sequences in the precipitated DNA in comparison to ChIP samples produced with control antibody (IgG) (Figure 3A). Immunoprecipitation with FLAG antibodies that used nuclear extracts from pcDNA3.1_PITX3_FLAG transfected cells resulted in enrichment of *MIP/AQP0* promoter sequences in the precipitated DNA in comparison to ChIP samples produced with control antibody (IgG)/same nuclear extracts as well as precipitations that utilized the same antibody (FLAG) but employed mock-transfected cells (Figure 3B). This enrichment for *MIP/AQP0* promoter sequences in the precipitated DNA was demonstrated by semi-quantitative PCR using specific *MIP/AQP0* and control primers (described in Materials and Methods) and calculated to be ∼2.2 times in experiments performed in native untransfected HLE cells and ∼4.5 times in assays that used transfected HLE cells; the observed differences were found to be statistically significant with P<0.05 based on t-test (Figure 3C and D). The chromatin immunoprecipitation and PCR-based enrichment analysis was repeated eight times using independently transfected cells with consistent results.

These experiments demonstrated the specific association of PITX3 with the *MIP/AQP0* promoter region *in vivo*.

### Mutations in bicoid sites located in the MIP/AQP0 promoter affect the activity of the promoter

To examine whether the conserved *bicoid* sites within the *MIP/AQP0* promoter are involved in regulation of its activity, we created several reporter constructs: *MIP656-bcd1,2*, which contained a 656-bp fragment of the *MIP/AQP0* wild-type promoter encompassing both *bicoid* sites and nucleotides from positions −597 to +59 in relation to the *MIP/AQP0* transcriptional start site inserted into a basic pGL3 plasmid containing the luciferase reporter gene; *MIP656-bcd1*, which contained a mutation in the *bcd2* site that changed the 5′-TAATCC-3′ sequence to 5′-TAATTT-3′, thus abolishing its interaction with wild-type PITX3 [27]; *MIP656-bcd2*, which contained a similar mutation in the *bcd1* site changing the 5′-GGATTA-3′ sequence into 5′-AAATTA-3′; and *MIP656-bcd0*, which included both of the above described mutations.

**Figure 4:**
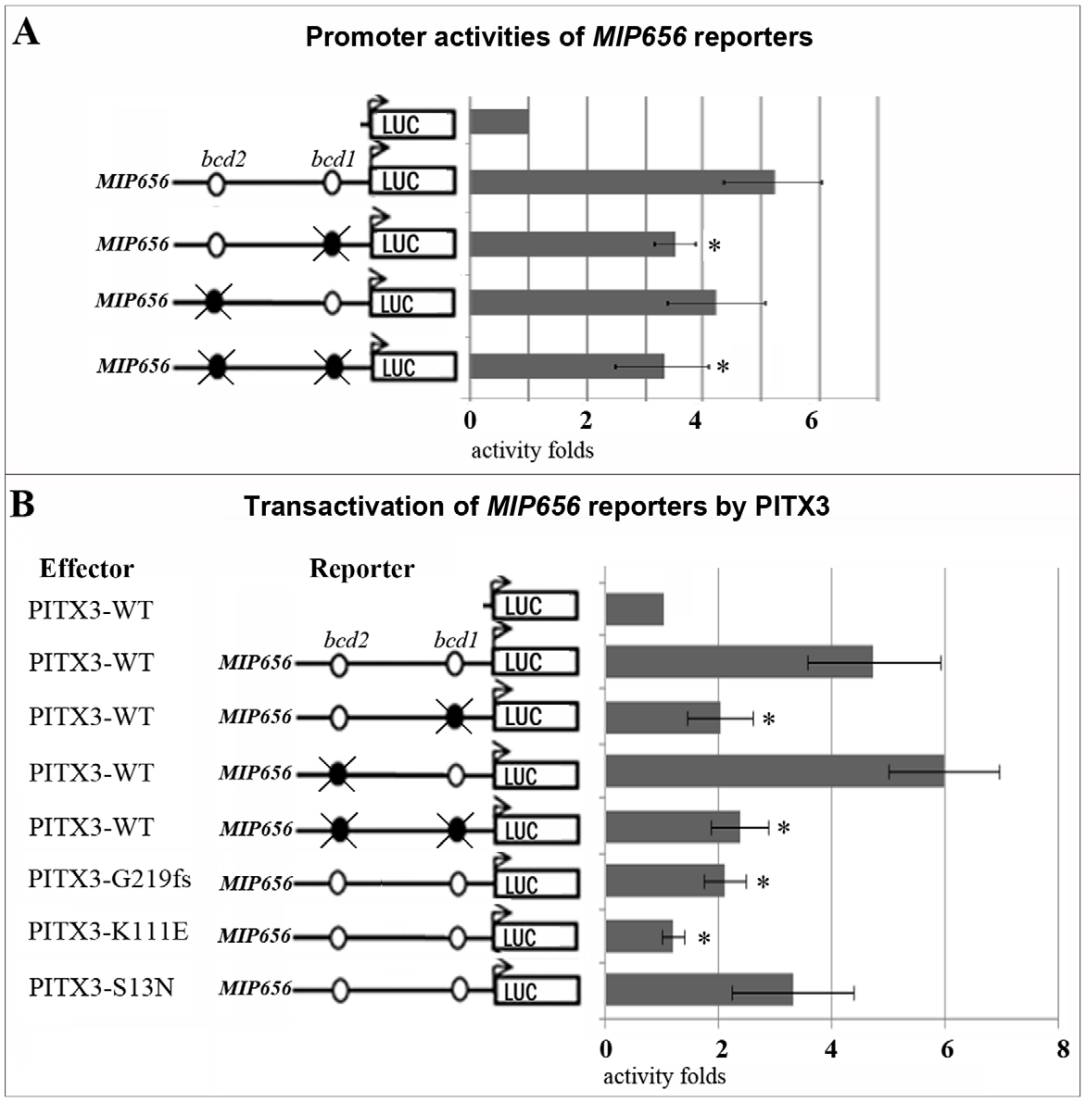
*MIP/AQP0* is activated by PITX3 via interaction with the proximal *bicoid* site, *bcd1*. **A.** Promoter activities of the *MIP656* reporters in human embryonic kidney cells **B.** Transactivation of *MIP656* reporters by PITX3 and its mutants in human embryonic kidney cells. Constructs and positions of *bicoid* sites are indicated on the left side. Wild-type *bcd1* or *bcd2* sites are depicted as open circles. Mutations (TAATCC to TAATTT substitutions) in *bcd1* or *bcd2* sites are depicted as dark circles with a strike-through. Student's paired t-Test with a one-tailed distribution was utilized to compare values. Experiments marked with asterisk (*) demonstrated a significant difference (P<0.001) in comparison to experiments performed with *MIP656* wild-type promoter (A) or *MIP656* wild-type promoter with PITX3-WT (B).

Reporter assays demonstrated a 5.2-fold upregulation of luciferase expression in the presence of the *MIP656-bcd1,2* promoter fragment in comparison to the empty vector in human embryonic kidney cells (Figure 4A). Mutations in either the *bcd1* or *bcd2* sites resulted in a decrease in *MIP/AQP0* promoter activity compared to the wild-type promoter: to 3.5-fold (67% of *MIP656-bcd1,2* activity; P<0.001) when the *bcd1* site was mutated, to 4.3-fold (83%; P = 0.014) when the *bcd2* site was abolished and to 3.4-fold (65%; P<0.001) when both sites were disrupted.

Based upon these results, both *bicoid* sites appear to be involved in regulation of *MIP/AQP0* expression with the proximal site, *bcd1*, playing a more significant role in its activation.

### PITX3 is capable of transactivating the MIP/AQP0 promoter via the bcd1 bicoid site

In order to investigate the effect of PITX3 on the transcriptional activity of the *MIP/AQP0* promoter, we performed cotransfection assays using the above described *MIP656-bcd1,2, MIP656-bcd1, MIP656-bcd2* and *MIP656-bcd0* reporter constructs and a *PITX3* expression plasmid.

Cotransfection of the *PITX3-WT* expression plasmid with the *MIP656-bcd1,2* luciferase reporter into human embryonic kidney cells resulted in a ∼4.8-fold normalized activation in comparison to the cotransfection of the *PITX3-WT* plasmid with the promoter-less reporter (Figure 4B). In contrast, cotransfection of the *MIP656-bcd1,2* luciferase reporter with an expression plasmid carrying *PITX3* mutants produced a ∼2-fold increase over the same control for the *G219f s* mutant (42% of wild-type activity; P<0.001), ∼3.1-fold for *S13N* (65%; P = 0.015) and no transactivation was observed for the *K111E* mutant. These results are consistent with the previously reported data on the residual activities of the corresponding mutant PITX3 forms [27].

We next examined the two *bicoid* sites present in the *MIP/AQP0* promoter for their role in this observed transactivation. The *PITX3-WT* expression plasmid and mutant *MIP/AQP0* promoter constructs, *MIP656-bcd1* (carrying mutant *bcd2* site), *MIP656-bcd2* (carrying mutant *bcd1* site) and *MIP656-bcd0* (carrying mutations in both *bicoid* sites) were cotransfected into human embryonic kidney cells and the resultant luciferase activities were compared to values observed in experiments involving cotransfection of wild-type *MIP/AQP0* promoter (*MIP656-bcd1,2*) and *PITX3-WT*. The transactivation of the *MIP656-bcd1,2* promoter by *PITX3-WT* decreased to ∼2-fold (42% of PITX3 induced wild-type promoter transactivation) when the mutation in the proximal *bicoid* site, *bcd1*, was introduced; increased to ∼6-fold (125%; P = 0.03) when the distal *bicoid* site, *bcd2*, was mutated; and produced ∼2.2-folds (46%; P<0.001) when both *bicoid* sites were disrupted (Figure 4B).

These data suggest that PITX3 is involved in activation of the *MIP/AQP0* promoter via its proximal *bicoid* site, *bcd1*.

### Knockdown of pitx3 affects formation of a DNA-protein complex associated with mip1 promoter sequences

**Figure 5:**
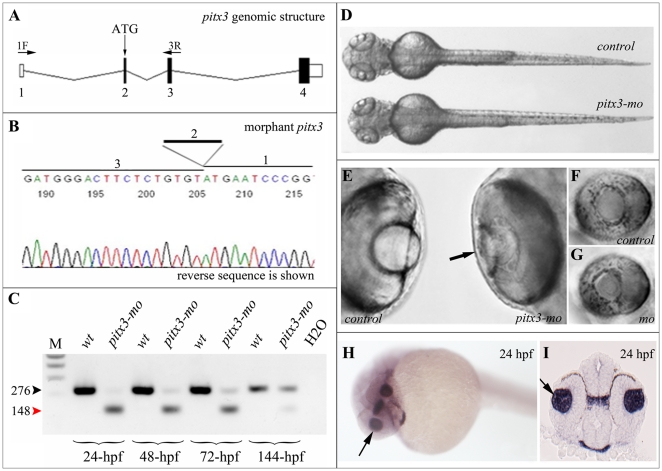
Injection of *pitx3* morpholino results in abnormal splicing of *pitx3* transcript and small lens phenotype. **A.** Schematic drawing of *pitx3* gene; position of initiation codon (ATG) and RT-PCR primers (1F and 3R) are indicated, exons are numbered. **B.** Sequencing of the *pitx3-mo* transcript generated with primers located in the first and third *pitx3* exons demonstrates absence of exon 2 in the resultant product. **C.** RT-PCR results obtained with *pitx3* 1F/3R primers using RNA extracted from embryos injected with control or *pitx3* morpholino. Please note a strong decrease in normal 276-bp product (black arrowhead) and presence of abnormal 148-bp product (red arrowhead) in *pitx3-mo* samples; hpf- hours post fertilization of analyzed embryos. **D–G.** Morphological phenotypes of zebrafish *pitx3-mo* embryos. In comparison to control embryos, a smaller head can be observed in *pitx3* morphants (72-hpf embryo is shown; D) as well as an obvious reduction in lens size (black arrow) at later stages (96-hpf embryos are shown; E–G). **H** and **I.** Expression of *pitx3* in the developing lens in 24-hpf embryos. Please note robust expression in the lens vesicle (arrows) as demonstrated by both whole mount (H) and section (I) in situ.

In order to efficiently disrupt *pitx3* gene expression in zebrafish and to be able to tightly monitor residual activity/knockdown level, we tested several splicing morpholino that were designed against *pitx3* intron-exon junctions. Unfortunately, none of these morpholinos produced the desired outcome, resulting in either no effect on *pitx3* splicing or highly abnormal phenotype due to toxicity/non-specific defects. Then we tested the previously reported translational morpholinos [9], [12] and discovered that the antisense morpholino reported by Dutta and coauthors [9] and designed against the sequence containing the translation initiation codon located in exon 2 of *pitx3* results in abnormal splicing of the *pitx3* transcript due to exon 2 skipping (Figure 5). This *pitx3* morpholino [9] matches the nucleotide sequence at positions +8 to +32 of exon 2 and therefore is located only 7-nt upstream of the intron 1/exon 2 acceptor site. In our experiments, we found this morpholino to be highly efficient in blocking normal splicing with the abnormal 148-bp product lacking exon 2 generated in *pitx3-mo* injected embryos versus the normal 276-bp product containing exon 2 seen in *control-mo* injected embryos (Figure 5B, C). The first potential initiation codon (for methionine) in the *pitx3-mo* transcript is located at position 28 of the pitx3 homeodomain and, as a result, the translation of this transcript will produce an abnormal protein lacking the N-terminal region and 45% of its homeodomain and, therefore, predicted to be nonfunctional.

An abnormal phenotype was detected in ∼95% of *pitx3-mo* injected embryos; early lethality (before 20-hpf) was observed in ∼20% of *pitx3-mo* and *control-mo* injected embryos. The *pitx3-mo* displayed a misshapen smaller head, jaw abnormalities and reduction in eye size due to progressive lens degeneration and retinal defects leading to a complete loss of lens by 7-dpf consistent with the previous reports [9], [12] (Figure 5D–G). Robust expression of *pitx3* is seen in lens vesicle at 24-hpf and it continues to be highly expressed during all stages of lens development [9]–[13; Figure 5H and I]. In addition to the strong lens expression, *pitx3* transcripts are also detected in the developing brain, craniofacial region and trunk musculature as previously described [9], [12], [13], [41], [42].

**Figure 6:**
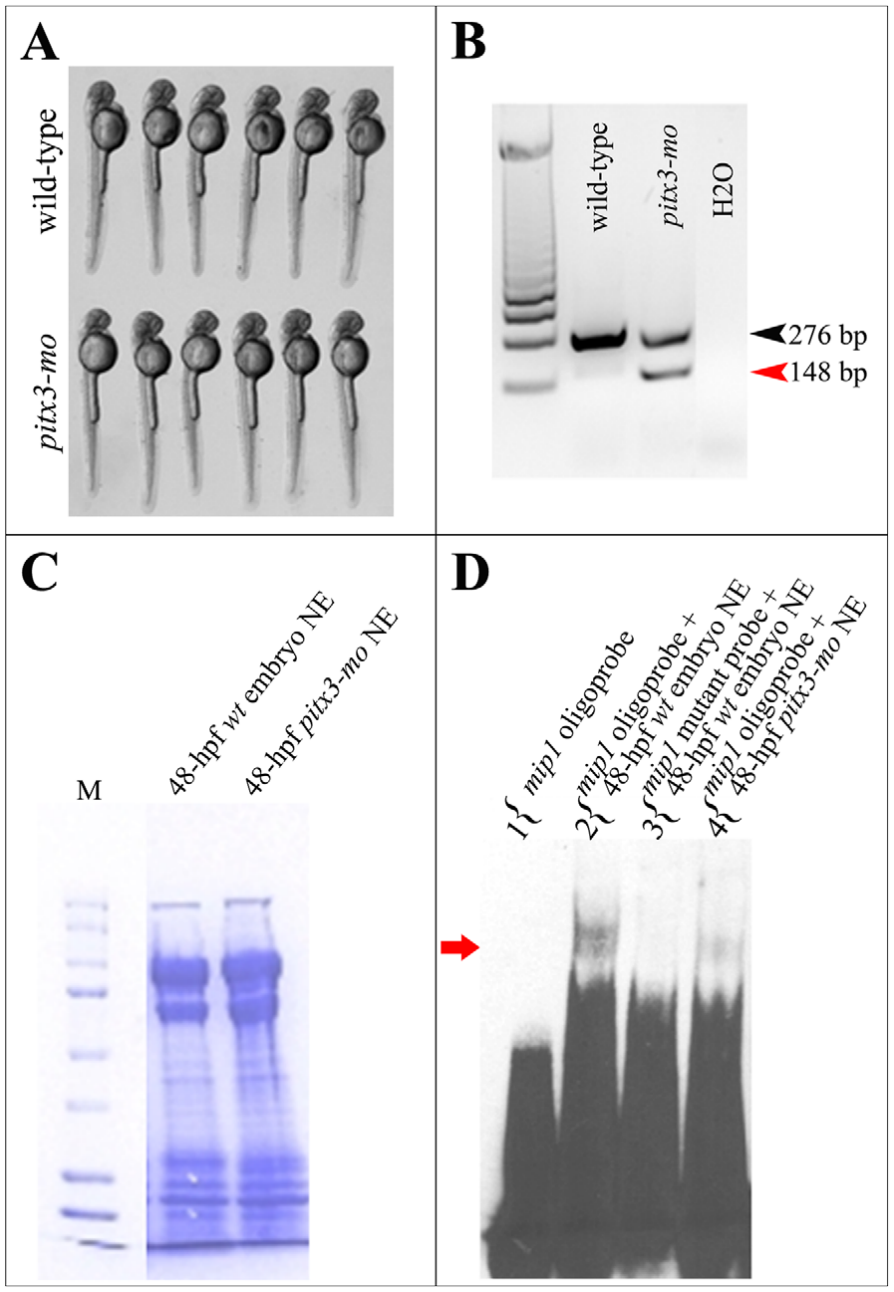
Formation of high molecular weight *mip1* promoter- protein complexes is dependent on pitx3 presence. **A.** 48-hpf wild-type and *pitx3* morphant embryos showing normal body length and morphology that were selected for EMSA experiments. **B.** Results of RT-PCR performed with RNA extracted from the pooled tail tissues from wild-type and *pitx3* morphant embryos shown in C. A sharp reduction in normal *pitx3* transcript (black arrowhead) and the presence of abnormally spliced product (red arrowhead) are evident in *pitx3* morphant embryos. **C.** Coomassie Blue R-250 stained polyacrilamide gel demonstrating equal protein concentration in nuclear extracts obtained from wild-type (lane 1) and *pitx3* morphant (lane 2) embryos that were used in EMSA experiments shown in A. **D.** Electrophoretic mobility shift assays (EMSA) show formation of a DNA-protein complex when an oligonucleotide corresponding to the −44/−76 region of zebrafish *mip1* promoter and nuclear extracts from 48-hpf wild-type zebrafish embryos are used. Please note a presence of a specific slow migrating complex, which is formed by wild-type *mip1* probe and proteins extracted from nuclei of 48-hpf wild-type zebrafish embryos (lane 2), absence of this complex in lane 3 when the same nuclear extracts were combined with a mutant *mip1* probe where the pitx3-binding *bicoid* site GGATTA was replaced by AAATTA, and sharp reduction of this complex in lane 4 containing a combination of a wild-type *mip1* probe and nuclear extracts obtained from *pitx3* morphants (red arrow).

Since we demonstrated above that PITX3 is capable of binding human *MIP/AQP0* promoter *in vivo*, further experiments were performed to establish if knockdown of *pitx3* would affect formation of protein-*mip1* promoter complexes in zebrafish. We injected zebrafish embryos with above described *pitx3* morpholino oligonucleotides that result in abnormal splicing of *pitx3* transcript. Embryos were harvested at 48-hpf and nuclear extracts from wild-type embryos and *pitx3* morphants were tested for their ability to bind a biotinylated DNA fragment containing the proximal *bicoid* site and corresponding to zebrafish −44/−76 *mip1* promoter region (Figure 6). The experiments were performed using nuclear protein extracts isolated from the upper trunk/head region of the 48-hpf wild-type and *pitx3* morphant embryos that displayed normal body length and morphology (Figure 6A) and demonstrated a normal presence (wild-type) or a significant reduction (*pitx3-mo*) in normal *pitx3* transcript based on RT-PCR analysis performed using RNA extracted from the lower trunk region of the same embryos (Figure 6B). For positive control, aliquots of nuclear extracts from wild-type and *pitx3* morphants were analyzed on 10% polyacrylamide gel followed by Coomassie Blue R-250 staining to assure equal protein concentration in both samples (Figure 6C). Two apparent slow-migrating complexes were formed that were evident at the top of the gel when EMSA was performed with nuclear extracts obtained from wild-type embryos. The formation of these complexes was abolished by introduction of a mutation into the *bicoid* site contained within the −44/−76 *mip1* promoter, which suggests that pitx3 is directly involved in DNA-binding of this fragment (Figure 6D). In addition to this, the slow-migrating complexes were significantly diminished when nuclear extract obtained from *pitx3* morphants were utilized (Figure 6D). Therefore the observed reduction in the intensity of the slow-migrating DNA-protein complexes correlates well with the residual amount of normal *pitx3* transcript in morphants in this experiment.

These data support our previous findings which demonstrate that pitx3 is a part of large complex occupying the *mip1* promoter in the developing zebrafish embryo.

### Knockdown of pitx3 affects mip1 expression in zebrafish embryos

**Figure 7:**
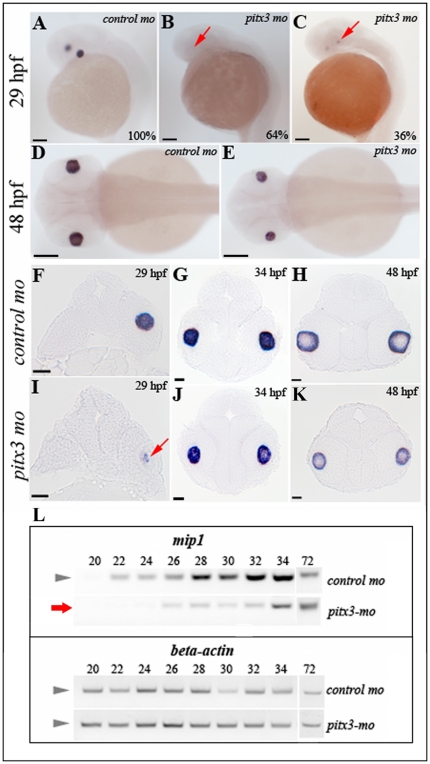
Analysis of *mip1* expression in *pitx3-mo* and control embryos via *in situ* hybridization and RT-PCR. **A, D, F-H.** Normal *mip1* expression in control-injected embryos at 29-, 34- and 48-hpf. **B, C, E, I–K.** Altered *mip1* expression is observed in *pitx3* morphants at 29-hpf with 64% of embryos demonstrating a complete absence of *mip1* expression (B) and the remaining larvae showing markedly reduced *mip1* expression (C and I). Reduced *mip1* expression is also observed in 34- and 48-hpf embryos (E, J, K). Red arrows show sites of expected *mip1* expression. Scale bars: A–E: 100 µM; F–L: 20 µM. **L.** Results of semi-quantitative RT-PCR showing reduced expression of *mip1* in *pitx3* morphants at early stages of development (red arrow).

Examination of *mip1* expression by *in situ* hybridization identified a specific and robust expression pattern in 100% of *control-mo* injected embryos (15/15), while a complete absence (9/14 or 64.3%) or a very low level (5/14 or 35.7%) of *mip1* expression was seen in *pitx3-mo* embryos at 29-hpf (Figure 7A–C, F, I). *mip1* expression is clearly observed in both control and *pitx3-mo* injected embryos at later stages but appears to be somewhat reduced in *pitx3* morphants (Figure 7 D, E, G, H, J, K).

Semi-quantitative RT-PCR analysis of *mip1* expression in the *pitx3* morphants and control-injected embryos confirmed a specific delay and decrease in *mip1* expression in *pitx3* morphants. The expression was initiated at ∼22-hpf in control injected embryos consistent with the start of lens fiber cell differentiation. The expression increased at later stages with the highest levels being detected in 48-hpf embryos and decreased levels by 72-hpf. In the *pitx3-mo* embryos, the first expression was observed in 26-hpf embryos with expression levels being noticeably lower in comparison to the *control-mo* injected larvae. The *mip1* expression in 28–34-hpf *pitx3* morphants continued to be reduced in comparison to control-injected larvae and but reached similar expression levels by 72-hpf (Figure 7L). Examination of *beta-actin* (loading control) demonstrated similar levels of expression between *pitx3-mo* and *control-mo* injected embryos (Figure 7L).

These experiments revealed a specific delay and decrease in expression of an important lens factor, *mip1*, in response to *pitx3* deficiency.

## Discussion

PITX3 is a homeodomain transcription factor that is essential to normal eye development in vertebrates. Yet, its direct downstream targets and mechanism of action are poorly understood. In this manuscript, we present identification of the first direct target of PITX3 during lens development, the major intrinsic protein of lens fibers, *MIP/AQP0*.

The *MIP/AQP0* promoter was identified via scanning of the human genome for regions containing conserved clusters of *bicoid* sequences and located in the proximity of known genes. Since the sequence, expression and function of PITX3 are conserved in vertebrates and *bicoid* sites are known to mediate its interaction with DNA, the conserved presence of these elements in a gene's promoter/regulatory region suggests that it may be regulated by PITX3. In addition to this, *MIP/AQP0* represents a logical downstream target of PITX3 because of its known role in lens development/function.

MIP/AQP0 is one of the most abundant proteins found in lens fibers where it acts as a water channel and adhesion molecule [43]–[46]. Mouse *Pitx3* and *Mip/Aqp0* display overlapping expression patterns during eye development as both genes are expressed in the developing primary and secondary lens fibers, with continued expression in adult organisms [1], [14], [47], [48]; expression of zebrafish *pitx3* precedes *mip1* in the developing lens [9]–[13; this manuscript] consistent with its proposed role in activation of *mip1* expression. Mutations in both *PITX3* and *MIP/AQP0* are implicated in congenital cataracts in humans [1]–[6], [49] and result in lens phenotypes in mice [7], [8], [18], [50]–[52]. Gene expression patterns as well as phenotypic abnormalities observed in mutant animals suggest an earlier appearance of *Pitx3* in comparison to *Mip/Aqp0*, which would also be consistent with *Mip/Aqo0* being a downstream target of Pitx3. Expression of the *Mip/Aqp0* transcript and protein is first detectable at mouse embryonic stage E11.25 in the ventro-temporal half of the lens vesicle concurrent with the initial stages of primary fiber cell differentiation and continues to be restricted to the lens differentiating primary and secondary fiber cells throughout adulthood [47], [48].

Transcriptional regulation of the *Mip/Aqp0* expression pattern is not yet well understood with several potential players discussed in the literature. Previous studies have shown that the human *MIP/AQP0* 5′ flanking sequence −253/+42 is sufficient for promoter activity in embryonic chicken lens epithelia primary cultures but is inactive in non-lens cells, suggesting that this region contains regulatory sequences required for the lens-specific expression of *MIP/AQP0* [53]. Ohtaka-Maruyama and colleagues reported that *MIP/AQP0* promoter fragments are protected in a DNase I footprinting assay performed with purified AP-2 and Sp1 and, therefore, the promoter is likely to interact with these factors [54]. Transgenic mice expressing *AP2-α* in lens fiber cells under the control of the αA-crystallin promoter were found to have reduced amounts of *Mip/Aqp0* in all fiber cells while expanded *Mip/Aqp0* expression was detected in the lens stalk of *AP2-α−/−* mice [55], [56]. Based on these observations and earlier reports [54], West-Mays and colleagues concluded that Ap2-α acts as a negative regulator of *Mip/Aqp0* expression and may, directly or indirectly, be responsible for its tight spatial/ temporal control [56]. In addition to these reports, *Mip* expression was shown to be triggered by treatment with FGF-2 and to accompany ERK1/2 and JNK activation in rat lens epithelia explants; treatment with specific ERK1/2 or JNK inhibitors resulted in abrogation of *Mip/Aqp0* expression in response to FGF-2 [57].

In this manuscript, we present evidence that the two evolutionarily conserved sequences containing *bicoid* sites within the *MIP/AQP0* promoter are capable of binding specifically to PITX3. Moreover, through chromatin immunoprecipitation assays we demonstrated that PITX3 is bound to the *MIP/AQP0* promoter *in vivo* in human lens epithelial cells. Analysis of the functional significance of this binding using luciferase reporter assays demonstrated that wild-type PITX3 is able to transactivate the *MIP/AQP0* promoter while mutant PITX3 forms showed reduced or absent transactivation ability. Further functional analysis utilizing site-specific mutations revealed the importance of the proximal *bicoid* site for the observed transactivation. The obliteration of the proximal *bicoid* site resulted in a statistically significant reduction of *MIP/AQP0* promoter activity as well as a decreased level of transactivation by PITX3. Finally, analyses performed in zebrafish embryos suggested that pitx3 is bound to the *mip1* promoter sequences during embryonic development and that *mip1* expression is altered in zebrafish *pitx3* morphants. At later stages of development (48–72-hpf), the expression of *mip1* in *pitx3* morphants appears to be largely unaffected, suggesting that regulation of *mip1* activity at these stages may be mainly controlled by other, *pitx3* pathway-independent, factors; pitx3 may also contribute to the recovery of *mip1* expression since increasing amounts of normal *pitx3* transcript can be observed in zebrafish embryos starting at 48-hpf due to weakening of the effects of morpholino injections (Figure 5C). Identification/development of permanent *pitx3* mutant lines is needed to allow more careful evaluation of the relationship between these factors.

Mutations in the human *MIP/AQP0* gene were shown to underlie various dominant forms of cataracts [49], [58]–[65]. To the best of our knowledge, only two of the reported *MIP/AQP0* mutations were explored for functional defects and a dominant-negative mechanism was suggested [58]. The dominant nature of *MIP/AQP0* mutations may also be explained by haploinsufficiency which would suggest that lens development is highly sensitive to dosage/timely expression of *MIP/AQP0*. The later possibility is further supported by the phenotype reported in the mouse carrying a null allele of *Aqp0* [54]. Deletion of mouse *Aqp0* was shown to result in cataracts at 3 weeks of age and at 24 weeks of age in homozygous and heterozygous mice, respectively. In heterozygous animals, the lens osmotic water permeability value was reduced to around 46% and the lens focusing power was significantly decreased in comparison to wild-type [52]. These findings demonstrated that a loss of one *Aqp0* allele, which presumably leads to reduced *Aqp0* expression, can be associated with lens abnormalities. Therefore, since *MIP/AQP0* represents an apparent transcriptional target of PITX3, the alteration of the *MIP/AQP0* expression in patients affected with *PITX3* mutations is likely to contribute to the lens phenotype observed in these individuals.

Further studies of the *MIP/AQP0* promoter will not only yield important insight into the transcriptional regulation of this critical lens differentiation factor but will also provide better understanding of the function of PITX3 and its interacting partners and allow for more specific identification of additional downstream targets of this ocular factor. Also, genetic screening of both *PITX3* and *MIP/AQP0* in human patients affected with ocular conditions may lead to an identification of synergistic or compensatory mutations/variants that may help to explain the considerable intra- and interfamilial phenotypic variability associated with mutations in either gene.
